# Plug-and-play inference for disease dynamics: measles in large and small populations as a case study

**DOI:** 10.1098/rsif.2009.0151

**Published:** 2009-06-17

**Authors:** Daihai He, Edward L. Ionides, Aaron A. King

**Affiliations:** 1 Department of Ecology and Evolutionary Biology, University of Michigan, Ann Arbor, MI 48109, USA; 2 Department of Statistics, University of Michigan, Ann Arbor, MI 48109, USA; 3 Department of Mathematics, University of Michigan, Ann Arbor, MI 48109, USA; 4 Fogarty International Center, National Institutes of Health, Bethesda, MD 20892, USA

**Keywords:** mechanistic model, iterated filtering, sequential Monte Carlo, measles, state-space model

## Abstract

Statistical inference for mechanistic models of partially observed dynamic systems is an active area of research. Most existing inference methods place substantial restrictions upon the form of models that can be fitted and hence upon the nature of the scientific hypotheses that can be entertained and the data that can be used to evaluate them. In contrast, the so-called *plug-and-play* methods require only simulations from a model and are thus free of such restrictions. We show the utility of the plug-and-play approach in the context of an investigation of measles transmission dynamics. Our novel methodology enables us to ask and answer questions that previous analyses have been unable to address. Specifically, we demonstrate that plug-and-play methods permit the development of a modelling and inference framework applicable to data from both large and small populations. We thereby obtain novel insights into the nature of heterogeneity in mixing and comment on the importance of including extra-demographic stochasticity as a means of dealing with environmental stochasticity and model misspecification. Our approach is readily applicable to many other epidemiological and ecological systems.

## 1. Introduction

The ability to forecast, understand and control the spread of infectious diseases increasingly depends on the capacity to formulate and test mathematical models capturing key mechanisms. Accordingly, there has been a great deal of recent interest in techniques that allow one to fit mechanistic models to time-series data (Gibson & Renshaw 1998; Finkenstädt & Grenfell 2000; Bjørnstad & Grenfell 2001; Gibson & Renshaw 2001; Grenfell *et al.* 2001, 2002; Roberts & Stramer 2001; Bjørnstad *et al.* 2002; Finkenstädt *et al.* 2002; Grenfell *et al.* 2002; Keeling & Rohani 2002; Clark & Bjørnstad 2004; Koelle & Pascual 2004; Streftaris & Gibson 2004; Beskos *et al.* 2006; Cook *et al.* 2007; Cauchemez & Ferguson 2008; Cauchemez *et al.* 2008; Ferrari *et al.* 2008; Keeling & Ross 2008). Recent advances in statistical algorithms and computational hardware have made it possible to tailor statistical methodology directly to questions of scientific interest and have expanded the range of models that can be confronted with data. However, the nonlinear stochastic dynamical models arising in the study of infectious disease dynamics have proved relatively recalcitrant. Even the simplest useful models of disease transmission are highly nonlinear, and stochasticity cannot be ignored even in the most predictable of disease systems. Complicating the picture further is the ubiquitous presence of non-stationarity, measurement error and unobserved (hidden, or latent) variables. Finally, models of interest are typically formulated in continuous time, whereas data are sampled at discrete and sometimes irregular intervals. The large body of methodological literature devoted to the topic of inference for these partially observed stochastic dynamic systems is testament to its position as an important and unresolved challenge at the interface of the fields of ecology, epidemiology, mathematics and statistics.

As general approaches, all of the methods mentioned earlier face severe limitations. Some must modify the model, discretizing time to the scale of observations (Finkenstädt & Grenfell 2000; Grenfell *et al.* 2001, 2002; Bjørnstad *et al.* 2002; Finkenstädt *et al.* 2002; Clark & Bjørnstad 2004; Koelle & Pascual 2004). They rely upon fortuitous congruence between some time scale of the process under study (e.g. infection generation time) and that of the sampling interval. These methods are computationally cheap, but can lead to severely biased conclusions (Glass *et al.* 2003). Others rely on approximations that are valid only in restricted circumstances. For example, Cauchemez & Ferguson (2008) adopt a diffusion approximation to enable successful implementation of a Markov chain Monte Carlo approach; this approximation breaks down in small populations. On the other hand, straightforward Bayesian approaches have been demonstrated for small populations with purely demographic stochasticity (Gibson & Renshaw 1998, 2001); however, these methods break down for large populations (Roberts & Stramer 2001; Beskos *et al.* 2006) or when environmental stochasticity is non-negligible (Bretó *et al.* 2009). Finally, exact methods based on numerical solution of the so-called master equations (Keeling & Ross 2008) are available, but limited to low-dimensional models and small populations. In summary, each of these approaches makes demands upon the form of the model and/or the nature of the data and thus necessitates scientifically irrelevant or even inappropriate assumptions that can interfere with the hypotheses of interest.

In this paper, we use a relatively new approach with none of the aforementioned limitations (Ionides *et al.* 2006, 2007, 2009; King *et al.* 2008; Bretó *et al.* 2009). In particular, the method works on continuous time models, in large or small populations, with demographic or environmental stochasticity or both. It has the important advantage of operation based only on simulation from the dynamic model, without a need for explicit expressions for transition probabilities. The latter property has been called *plug-and-play* (Bretó *et al.* 2009). Plug-and-play inference methodology provides a powerful tool to enable carrying out data analysis via flexible, scientifically motivated classes of models. In particular, plug-and-play is extremely useful when one wishes to entertain multiple working hypotheses translated into multiple mechanistic models. Two properties analogous to plug-and-play have been considered in the contexts of optimization and complex system theory.

- In optimization theory, methods requiring only evaluation of the objective function have been called *gradient-free* (Spall 2003). In such methods, computer code to evaluate the objective function can be plugged into the optimizer. This is particularly useful in situations in which the objective function is a complex, and possibly stochastic, function for which the analytic calculation of derivatives is difficult or impossible (Kocsis & Szepesvári 2006).
- In the analysis of complex systems, methods that are based solely on simulations from computer codes representing a model have been called *equation-free* (Kevrekidis *et al.* 2004). A typical goal of such investigations is to determine the relationship between macroscopic phenomena (e.g. phase transitions) and microscopic phenomena (e.g. molecular interactions).

These analogies suggest that plug-and-play is a subject whose importance extends beyond our present topic of inference for partially observed stochastic dynamic systems. The conceptual commonality is that all these methodologies can be applied to models specified as ‘black-box’ computer codes. This enables flexible model development and therefore avoids confounding methodological issues with scientific hypotheses. It also facilitates both the comparison of alternative explanations and the transfer of methodological expertise between different systems. The price of plug-and-play is primarily in terms of computational effort: when properties of a model are available in closed form, computationally more efficient methods typically exist. As computational capabilities continue to grow, the ease with which new scientific questions can be asked and answered via plug-and-play methodology will make such techniques increasingly attractive.

We illustrate our new approach via a case study of measles transmission dynamics. A focus on this well-studied disease allows us to compare our conclusions with those derived previously by other means. Further, we demonstrate that our analysis leads to fresh insights not readily available via previous analyses. Measles has occupied a central place in mathematical epidemiology owing to its relatively straightforward diagnosis, the lifelong immunity following infection and the availability of high-quality case report data. Models that assume mass-action transmission on the scale of communities do remarkably well at reproducing the disease dynamics, particularly in large populations (Bailey 1955; Bartlett 1960; Fine & Clarkson 1982), although recent work has suggested that deviations from mass action are detectable in time-series data (Bjørnstad *et al.* 2002). Moreover, models incorporating purely demographic stochasticity have been deemed adequate to reproduce observed dynamical patterns. We revisit these questions by fitting a family of models that incorporate deviations from the mass-action assumption together with both environmental and demographic stochasticity. We fit these models to time-series data from both large and small populations. A comparison of results across community sizes indicates that there is little evidence in the data for deviations from mass-action transmission, at least as it has been modelled previously. On the other hand, our results do suggest a role for heterogeneity in transmission as a function of population size. With respect to the nature of stochasticity in this system, our results indicate a clear role for extra-demographic variability. Indeed, they suggest that failure to allow for extra-demographic variability may lead to severe biases in estimates of key parameters. These insights are possible because of the flexibility of the inference machinery, which also makes our approach more readily applicable to other, less exhaustively analysed, systems (e.g. King *et al.* 2008).

## 2. Methods: inference machinery, data and models

Consider a time series *y*_1:*N*_ ≡ *y*_1_, … ,*y*_*N*_, consisting of *N* observations made at times *t*_1_, … ,*t*_*N*_. A stochastic model for *y*_1:*N*_ implies a joint density *f*(*y*_1:*N*_|*θ*) given a vector of unknown parameters *θ*, where *θ* is in ℝ^*n*_*θ*_^. Corresponding conditional densities for *y*_*n*_ given *y*_1:*n*–1_ are written as 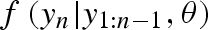. Via the factorization 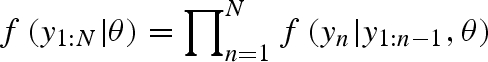, the log-likelihood function is given by 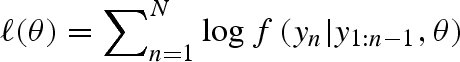. Sequential Monte Carlo (Doucet *et al.* 2001; Arulampalam *et al.* 2002; Cappé *et al.* 2007) provides a standard method to obtain the log likelihood for stochastic dynamic systems. In our terminology, *stochastic dynamic system* is synonymous with *partially observed Markov process* (Ionides *et al.* 2006; Bretó *et al.* 2009); in the statistical literature, these systems are known as *state-space models* (Shumway & Stoffer 2006). Likelihood evaluation via sequential Monte Carlo has the feature that only simulations of sample paths are required; one need not have explicit forms for transition probabilities. That is, it has the plug-and-play property. Both Bayesian (Liu & West 2001; Toni *et al.* 2009) and frequentist (Ionides *et al.* 2006; Poyiadjis *et al.* 2006) approaches to plug-and-play likelihood-based inference via sequential Monte Carlo have been proposed. We adopt the maximum-likelihood approach of Ionides *et al.* (2006, 2009), which enables statistically efficient inference for general nonlinear stochastic dynamical systems. In contrast, the approximations developed by Liu & West (2001) are not statistically efficient, and lead to additional bias and variance in the resulting parameter estimates (Storvik 2002; Newman *et al.* 2008). The popularity of the artificial parameter evolution method of Liu & West (2001) may be due more to the convenience of its plug-and-play property than to its statistical properties. Various refinements and variations in the vein of the Bayesian methodology of Liu & West (2001) have been studied (Polson *et al.* 2008 and references therein); however, these typically do not posses the plug-and-play property.

We used the implementation of iterated filtering contained in the software package Pomp (King *et al.* 2009) written for the *R* statistical computing environment (R Development Core Team 2006). This implementation follows the algorithm described by King *et al.* (2008, supplementary information therein). Heuristics and diagnostics, together with a discussion of computational issues, can be found in Ionides *et al.* (2006); a theoretical treatment is given by Ionides *et al.* (2009).

For complex dynamical systems, not all questions that one might wish to ask will be clearly answered by the available data. Typically, a question posed of the dynamic system is formalized as a hypothesis about a combination of parameters in a model for the system. If the likelihood function does not discriminate well between different values of some parameter combination, this parameter combination is said to be poorly identified. In such a case, one might proceed by making additional assumptions: adding constraints typically improves parameter identifiability at the cost of potential bias in the estimates of other parameters. In §3, we focus on questions the data are able to answer without further assumptions. A basic tool for this is the profile likelihood function, in which one parameter is varied over a range of values, while the likelihood is maximized over all remaining parameters (Barndorff-Nielsen & Cox 1994; Hilborn & Mangel 1997). In practice, we work with a smoothed estimate of the likelihood surface calculated via Monte Carlo (Ionides 2005).

**Figure 1.**
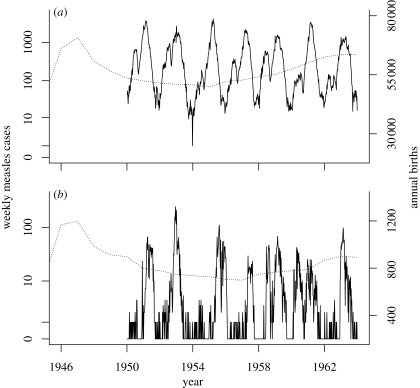
Weekly reported measles cases (solid line) and annual birth rate (dotted line) for (*a*) London and (*b*) Hastings.

The data we analyse consist of weekly measles case reports on 20 representative communities with population sizes ranging from 2000 to 3 000 000. The towns and cities were selected from 954 urban locations for which case reports have been compiled (Grenfell & Bolker 1998); specifically, we chose the largest 10 cities by population and sampled 10 more towns at random. Figure 1 shows the case reports and annual birth rates for two of these: London and Hastings. These data and their limitations have been discussed previously (Fine & Clarkson 1982; Bjørnstad *et al.* 2002). In particular, although measles became a notifiable disease in England and Wales in 1940, we follow Fine & Clarkson (1982) by starting our analysis in 1950 to avoid the disruptions caused by the second world war and the transition period following the introduction of the National Health Service in 1948. From 1950 onwards, it is believed that reporting was carried out fairly consistently, with between half and two-thirds of all cases being counted.

**Figure 2.**
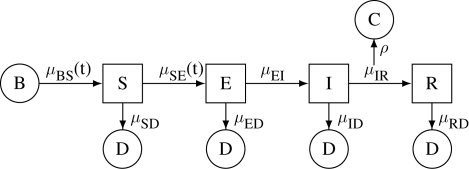
Flow diagram for measles. The population is divided into four compartments: S, susceptible; E, exposed and infected but not yet infectious; I, infectious; R, recovered and immune. Births enter S at the rate *μ*_BS_(*t*), and all individuals have a mortality rate *μ*_SD_ = *μ*_ED_ = *μ*_ID_ = *μ*_RD_ = *m*. The case reports, C, count infected individuals with probability *ρ*. Since diagnosed cases are treated with bed-rest and hence removed, infections are counted upon transition to R.

A basic susceptible–exposed–infectious–recovered (SEIR) model for measles divides the population into those individuals susceptible to infection, exposed (i.e. infected but not yet infectious), infectious and removed (i.e. quarantined or recovered and subsequently immune). The model is specified by describing the rates at which individuals move between compartments (Jacquez 1996; Matis & Kiffe 2000); figure 2 shows the transitions for the SEIR model of measles. A diagram such as figure 2 can be unambiguously interpreted as a deterministic system, a system with demographic stochasticity or a system with both demographic and extra-demographic stochasticity (Bretó *et al.* 2009). We give the equations for each of these interpretations in appendix A. Here, we will employ the stochastic interpretation, with both demographic and extra-demographic stochasticity, described in box 1 (in appendix A). The transition rates in a model such as figure 2 may, in general, depend on the state of the system and/or covariates. We specify the force of infection as

2.1
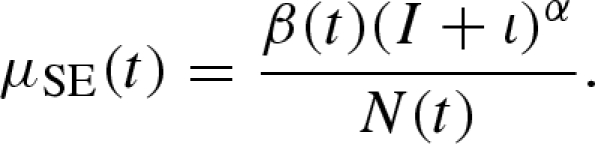


Here *β*(*t*) is the transmission rate; *ι* the mean number of infectives visiting the population at any given time; *α* a mixing parameter, with *α* = 1 corresponding to homogeneous mixing (Liu *et al.* 1986; Bjørnstad *et al.* 2002), and *N*(*t*) the population size, treated as known via interpolation from census data. Since transmission rates are closely linked to contact rates among children, which are higher during school terms (Schenzle 1984; Keeling & Grenfell 2002; Bauch & Earn 2003; Conlan & Grenfell 2007), we assume that *β*(*t*) reflects the pattern of British school terms and holidays. Specifically, we take
2.2
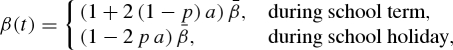

where *p* = 0.759 is the proportion of the year taken up by the school term, *β̄* the mean transmission rate and *a* the relative effect of school holidays on transmission. For ease of interpretation, it is sometimes convenient to reparameterize *β̄* in terms of the annual average basic reproductive ratio, *R*_0_, defined as the expected number of secondary infections engendered by an infective introduced into a fully susceptible population. When the duration of infection is much shorter than life expectancy, *R*_0_ ≈ *β̄*/*μ*_IR_; here we employ a modification of this formula, given in appendix A.

A novel feature of our model is that we add white noise with intensity *σ*_SE_ to the transmission process. Some empirical support for the choice of white (i.e. temporally uncorrelated) noise is given by Lande *et al.* (2003). This variability in the rates allows the possibility of over-dispersion (McCullagh & Nelder 1989; Bretó *et al.* 2009). It is discussed at more length, together with a full specification of all the model equations, in appendix A.

The other transition rates are specified as follows: *μ*_EI_ is the rate at which exposed individuals become infectious, thus *μ*_EI_^−1^ is the mean latent period; *μ*_IR_ is the recovery rate; *μ*_SD_ = *μ*_ED_ = *μ*_ID_ = *μ*_RD_ = *m* denotes a constant per-capita death rate, ignoring the negligible effect of disease-induced mortality; *μ*_BS_(*t*) is the per-capita rate of recruitment of susceptibles, depending on the birth rate *b*(*t*), which is assumed to be known via interpolation from birth records. Births enter the susceptible class with a delay *τ* corresponding to the age at which children enter the high-risk school-age population. We also consider a cohort-entry effect that reflects the fact that a large cohort of first-year students—the majority of them serologically naive—enters the schools each autumn (Schenzle 1984; He 2006). Specifically, a fraction *c* of recruits into the susceptible class enter on the school admission day, and the remaining fraction (1–*c*) enter the susceptible class continuously. The cohort effect provides a parsimonious and mechanistically plausible alternative to previous suggestions that the transmission rate *β*(*t*) may increase following the start of the academic year (Fine & Clarkson 1982; Grenfell *et al.* 2002). The reporting process is taken to be on over-dispersed binomial, with reporting rate *ρ* and overdispersion parameter *ψ* (appendix A, equation (A 6)). Quantities appearing in the model are summarized in table 1.

**Table 1.** List of symbols used in the paper.

symbol	description	units	reference
*μ*_*ij*_	per-capita rate of transition from compartment *i* to *j*	yr^−1^	figure 2, box 1
*σ*_*ij*_	white-noise intensity on *i* → *j* transition rate	yr^1/2^	box 1
LP	latent period	day	equation (A 7)
IP	infectious period	day	equation (A 7)
*ι*	mean number of visiting infectives	—	equation (2.1)
*α*	mixing exponent	—	equation (2.1)
*β*(*t*)	transmission rate	yr^−1^	equations (2.1) and (2.2)
*N*(*t*)	population size	—	equation 2.1
*a*	amplitude of seasonality	—	equation 2.2
*b*(*t*)	birth rate	yr^−1^	equation (A 5)
*c*	cohort entry fraction	—	equation (A 5)
*τ*	recruitment delay	yr	equation (A 5)
*m*	mortality rate *μ*_*jD*_ for *j* ∈ {S, E, I, R}	yr^−1^	figure 2
*ρ*	reporting probability	—	equation (A 6)
*ψ*	reporting overdispersion	—	equation (A 6)
*R*_0_	basic reproductive ratio	—	—

## 3. Results

Maximum-likelihood parameter estimates for our sample of 20 communities are presented in table 2. These estimates are for the model in §2 with no constraints placed on the parameters. The variation in the parameter estimates between populations has two sources: it may represent differences in the population dynamics of measles between communities, or it may be due to weak identifiability of some combinations of parameters. We disentangle these two explanations via a detailed investigation of two of them, London and Hastings. However, table 2 does reveal some consistent patterns that are robust across the population samples and are therefore not strongly affected by identifiability issues. These patterns are valuable for indicating model features about which the data under investigation are informative without the incorporation of additional assumptions based on prior analyses.

**Table 2.** Point estimates for 20 representative communities. *N* is 1950 population in thousands; LP and IP are the latent and infectious periods, respectively, in days, calculated from *μ*_EI_ and *μ*_IR_ as described in equation (A 7) of appendix A; other parameters and their units are as specified in table 1; the parameters *τ* = 4 yr and *m* = 0.02 yr^−1^ were not estimated. Maximized log-likelihood values are presented in the electronic supplementary material. The bottom row gives the Spearman rank correlation of the columns with *N* with * denoting significance at the 5 per cent level, ** the 1 per cent level and *** the 0.1 per cent level.

	*N*	*α*	*σ*_SE_	*R*_0_	LP	IP	*a*	*ι*	*c*	*ρ*	*ψ*
Halesworth	2	0.95	0.075	33	7.87	2.28	0.38	0.01	0.55	0.75	0.64
Lees	4	0.97	0.060	28	7.28	1.93	0.16	0.03	0.62	0.60	0.69
Mold	6	1.04	0.054	21	5.93	1.78	0.27	0.01	0.44	0.13	2.87
Dalton	11	0.99	0.078	28	5.48	1.98	0.20	0.04	0.42	0.46	0.82
Oswestry	11	1.04	0.070	53	10.29	2.72	0.34	0.03	0.26	0.63	0.48
Northwich	18	0.93	0.069	23	8.07	2.55	0.30	0.05	0.39	0.78	0.40
Bedwellty	29	0.94	0.061	25	6.82	3.03	0.16	0.04	0.35	0.31	0.95
Consett	39	1.01	0.071	36	9.07	2.66	0.20	0.07	0.31	0.65	0.41
Hastings	66	1.00	0.096	34	7.00	5.44	0.30	0.19	0.33	0.70	0.40
Cardiff	245	1.00	0.054	34	9.86	3.09	0.22	0.14	0.27	0.60	0.27
Bradford	294	1.00	0.053	36	10.80	3.22	0.25	0.27	0.30	0.60	0.19
Hull	302	0.97	0.064	39	9.18	5.46	0.22	0.14	0.27	0.58	0.26
Nottingham	307	0.98	0.038	23	5.72	3.69	0.16	0.17	0.34	0.61	0.26
Bristol	443	1.01	0.039	27	6.19	4.94	0.20	0.44	0.34	0.63	0.20
Leeds	510	1.00	0.078	48	9.48	10.92	0.27	1.25	0.59	0.67	0.17
Sheffield	515	1.02	0.043	33	7.23	6.38	0.31	0.85	0.23	0.65	0.17
Manchester	704	0.97	0.055	33	11.11	6.94	0.29	0.59	0.36	0.55	0.16
Liverpool	802	0.98	0.053	48	7.90	9.80	0.30	0.26	0.19	0.49	0.14
Birmingham	1118	1.02	0.056	35	11.23	7.90	0.31	1.09	0.61	0.56	0.19
London	3390	0.98	0.088	57	13.14	12.51	0.55	2.90	0.56	0.49	0.12
*r*		0.14	−0.27	0.44	0.46*	0.94***	0.26	0.94***	−0.16	−0.16	−0.93***	

**Figure 3.**
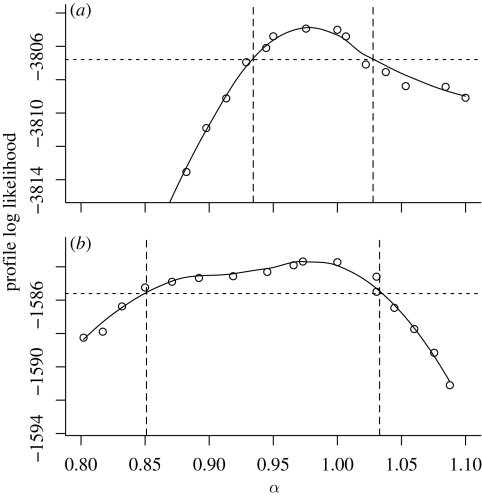
Profile likelihood analysis of the mixing parameter, *α*, for (*a*) London and (*b*) Hastings. The solid black lines show the estimated profile log likelihood, derived from the Monte Carlo point estimates shown as circles. The dashed lines construct approximately 95 per cent CIs of (0.93, 1.03) for London and of (0.85, 1.03) for Hastings.

In table 2, the homogeneity parameter *α* is consistently found to be close to 1. At first glance, this may suggest that there is little evidence for inhomogeneity of transmission in the data. Profile likelihood plots over *α* for London and Hastings are presented in figure 3. We see that the statistical uncertainty in the estimates for these two places is comparable to the geographic variability in the point estimates in table 2 (mean ± s.d. of 0.99 ± 0.03). This finding confirms the suggestion by Glass *et al.* (2003) that such exponents may be artefacts of the time discretization employed in earlier approaches (Bjørnstad *et al.* 2002; Grenfell *et al.* 2002). We can conclude that the phenomenon of inhomogeneous mixing—inasmuch as it plays a dynamic role—is best captured in some other way.

The imported infection parameter *ι* is well identified in these data. There is a strong log-linear relationship between *ι* and *N*, consistent with the earlier findings of Bjørnstad *et al.* (2002) and with the results obtained by Xia *et al.* (2004), who analysed a much more extensive dataset using a gravity model. The evidence for such a relationship is stronger here than in a previous empirical investigation (Finkenstädt *et al.* 2002, fig. 6).

**Figure 4.**
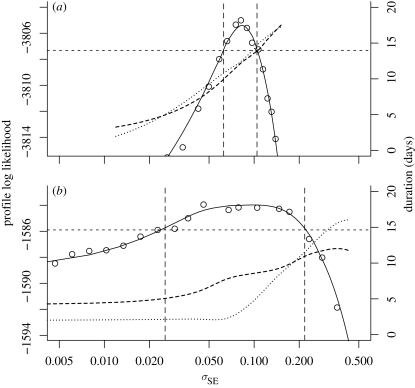
Profile analysis of extrademographic variability, *σ*_SE_, for (*a*) London and (*b*) Hastings. The solid black curve shows the estimated profile log likelihood, derived from the Monte Carlo point estimates shown as circles. The dashed lines construct approximately 95 per cent CIs for *σ*_SE_. Corresponding profile estimates of the mean IP and mean LP are shown with dotted and dashed curves, respectively, with units given on the right-hand axis. The figure is calculated with the constraint *α* = 1. Dotted lines, IP; dashed lines, LP.

The extra-demographic stochasticity parameter *σ*_SE_ is consistently found to be around 0.06 (0.063 ± 0.015). The profile likelihood plots in figure 4 also indicate that this parameter is fairly well estimable, and the data strongly argue against the choice *σ*_SE_ = 0. Figure 4 suggests that a role for extra-demographic stochasticity is not only strongly indicated for large cities such as London but also clearly evidenced in smaller populations for which one might expect demographic stochasticity to dominate. One interpretation of this is that models with purely demographic stochasticity (i.e. all discrete-population continuous-time dynamic models that have been previously fitted to disease data) fail to capture an extra-demographic source of variability that has an important dynamic role. *A priori* exclusion of the possibility of such additional variability can lead to spurious confidence in the conclusions of the analysis (McCullagh & Nelder 1989) and to biased parameter estimates. Figure 4 shows that, in the absence of extra-demographic stochasticity, estimates of the latent and infectious periods are substantially lower when one allows for such stochasticity. This appears to arise because short transition times lead to fewer individuals in compartments *E* and *I* on average and therefore to a larger dynamic role for demographic stochasticity. In other words, the model compensates for the lack of extra-demographic stochasticity by attempting to increase the intensity of demographic stochasticity.

Each parameter of the model has an interpretation in terms of the basic biology of transmission and infection at the level of individual hosts. To the extent that the model is a faithful description of the population-level processes, one expects that parameter estimates should agree closely with estimates based on individual-level observations. When discrepancies occur, they are evidence for model misspecification or oversimplification. Strikingly, table 2 shows a strong log-linear relationship between population size and infectious period and, to a lesser extent, latent period. Since there is no evidence that the natural history of measles varies with population size, this relationship is indicative of such oversimplification. Figure 1 suggests that this simple model fails to account adequately for heterogeneity in transmission. Specifically, the model calls for short infectious periods and high transmissibility *β* to reproduce the explosive epidemics and interepidemic fadeouts typical of small communities. The data from large populations, in contrast, represent the aggregate of many local epidemics, in each of the many schools and local communities contained in a large conurbation. In these aggregated data, fadeouts are rare and the epidemic curve is generally shallower. The oversimple model can reproduce these features only by extending the infectious period and reducing transmissibility. Moreover, while the infectious period estimated in the small populations (2–4 days) is consistent with evidence from household studies (Hope Simpson 1948, 1952; Bailey 1956), the longer infectious periods estimated in the large populations are not. Previous studies have noted that deterministic versions of the mass-action model analysed here do a remarkably good job of qualitatively reproducing the dynamics of measles in large communities (Earn *et al.* 2000). Our analysis shows, however, that when both continuous time dynamics and stochasticity are taken into account, data from small populations afford a less-distorted view of the disease dynamics, at least when viewed through the lens of relatively simple models.

The basic reproductive ratio of measles is conventionally held to lie between 14 and 18 (Anderson & May 1991). The values of *R*_0_ in table 2 are variable but consistently high relative to previous estimates (mean ± s.d. of 34.7 ± 10.1 over 20 communities). Profile likelihoods over *R*_0_ for London and Hastings (see the electronic supplementary material) yield approximately 95 per cent confidence intervals (CIs) of (37, 60) and (28, 74), respectively. Earlier empirical work on transmission dynamics (Bjørnstad *et al.* 2002) also led to a relatively high value of *R*_0_ = 29.9 for London. Bjørnstad *et al.* (2002) were cautious about the interpretation of this result because of concerns about the effects of the discrete-time methods employed. We can more confidently say that these higher values of *R*_0_ are a genuine feature of the continuous-time dynamics, at least under the assumption of homogeneous mixing and exponentially distributed latent and infectious periods. Changing the distribution of the latter to a more general gamma distribution (Keeling & Grenfell 1997; Lloyd 2001*b*; Wearing *et al.* 2005) decreases the estimated 95 per cent CI of *R*_0_ to (25, 41) for London (see the electronic supplementary material, figure S6). Though this is a substantial quantitative change, it does not alter the qualitative conclusion that homogeneous population models require high values of *R*_0_ in order to be consistent with the data.

## 4. Discussion and conclusion

A major goal of biomathematical modelling is to seek conceptual simplicity in the face of biological complexity. In general, the questions of interest should dictate the form and complexity of the model used to address them. In the present context in particular, the appropriate degree of aggregation over population inhomogeneities (such as spatial, socio-economic, genetic and age variation) may depend on the goals of the investigation (Levin *et al.* 1997; May 2004). For measles, analyses based on models of homogeneous mixing populations have proved useful in the study of seasonality (Fine & Clarkson 1982; Bjørnstad *et al.* 2002; Ferrari *et al.* 2008) and effects of climate drivers (Lima 2009). Metapopulations of weakly coupled homogeneously mixing populations are central to current understanding of spatio-temporal disease dynamics (Grenfell *et al.* 2001; Xia *et al.* 2004). In addition, such models can yield insight into the fundamental predictability of disease outbreaks (Stone *et al.* 2007) and have been found adequate to predict the effects of variation in birth rate and some dynamical features associated with the institution of vaccination programmes (Earn *et al.* 2000). Homogeneous mixing models were also the basis of early work on local extinctions and vaccination strategies (Bartlett 1957), though age structure and infectious period distribution may have substantial roles to play in these questions (Schenzle 1984; Keeling & Grenfell 1997; Lloyd 2001*b*; Conlan *et al.* submitted). In particular, estimates of the parameter *R*_0_ are known to be highly sensitive to assumptions on age structure (Wallinga *et al.* 2001). Interpretations of homogeneous mixing models must be made in the context of their limitations.

Clinical and household studies of infectious diseases yield information regarding the transmission and progression of infection on the scale of individuals or families (Hope Simpson 1948, 1952; Bailey 1956). Such studies are complemented by data on disease prevalence or incidence on larger spatial scales. The recent development of likelihood-based approaches capable of dealing with the full spectrum of unavoidable complexities (Cauchemez & Ferguson 2008; Cauchemez *et al.* 2008; King *et al.* 2008)—unobserved variables, measurement error, process noise, nonlinearity, non-stationarity and covariates—means that we can now view a disease from the population point of view with something like the same clarity that we have for many years been able to view individual infections. Specifically, although we have long understood how to describe the population dynamics of infectious diseases using mathematical models, we have only recently gained the ability to fit these models to data using statistically sound methods. When biological quantities estimated in this way agree with those estimated from clinical or household studies, it can be interpreted as confirmation of the assumptions embodied in the model. Disagreement between the small- and large-scale points of view, however, raises interesting scientific questions. In the present study, we find broad agreement with previous studies concerning many of the model's parameters. On the other hand, we find significant departures with respect to three important quantities—*R*_0_, the infectious period and the intensity of extra-demographic stochasticity.

The quantity *R*_0_ is central in epidemiological theory because it has interpretations in terms of so many quantities of interest, including mean age of first infection, mean susceptible fraction, exponential-phase epidemic growth rate and vaccination coverage required for eradication. It is important to realize that the conjunction of these interpretations occurs only in the context of very simple models. Simple models necessarily lack flexibility. In reality, these interpretations diverge owing to heterogeneities in age, spatial location, host genetics, etc. It is therefore unrealistic to expect that estimates of *R*_0_ (or any other single quantity) derived from fitting a simple model to one sort of data should agree with estimates derived from other data sources. Rather, the key question should be: which biological interpretations are relevant in the context of the data used to inform the model? From a statistical point of view, this corresponds to the question: to what features of the data are the parameter estimates sensitive? Our estimate of the basic reproductive ratio, *R*_0_, like those of earlier studies focusing on aggregated case-count time series, is somewhat greater than estimates based on serological surveys and age-stratified incidence data. It is possible that this reflects the sensitivity of the time-series-derived estimates of *R*_0_ to peak incidence. Specifically, peak incidence strongly influences the estimates of both *R*_0_ and reporting rate *ρ*, but since *ρ* is well identified by other features of the data (namely, the long-run cumulative incidence), we suspect that the model requires a high *R*_0_ to match the observed peak epidemic case counts. Alternatively, the high *R*_0_ estimates may reflect the sensitivity of estimated *R*_0_ to early phase epidemic growth. If this is in fact the case, our estimates of *R*_0_ may more accurately reflect contact rates among the core group of school-age children than they do those of the population at large. In this case, the model is effectively extrapolating these rates to the adult population, about which these data have little direct information since so few adult cases occur. In contrast, from this point of view, the interpretation of *R*_0_ in terms of its definition as mean number of secondary infections in a fully susceptible population is hopelessly extrapolated. Likewise, the interpretation of *R*_0_ in terms of mean age of first infection is unjustified, since it necessarily depends on the age structure of transmission, which is not part of the model. Perhaps the most direct information pertaining to mean age at first infection in these data comes from the lag between changes in birth rate and their subsequent effects on incidence. This lag is explicitly captured in our model by the delay, *τ*, between birth and recruitment into the susceptible pool.

Since the comparatively high estimate of *R*_0_ does not appear to be a mere artefact of time discretization, the question remains as to why the population-level data suggest a more communicable disease than do the individual-level data. Existing estimates of *R*_0_ are sensitive to assumptions about the age structure of transmission that have not yet been fully resolved (Wallinga *et al.* 2001). Perhaps transmission in schools is relatively more effectual than within households. Alternatively, it may be that this discrepancy points to a need for a better description of infectious- and/or latent-period distribution, of the disease's age structure or both. In our results, the departure of the estimated latent and infectious periods from plausible values obtained from household studies grows with city size, and so the most likely explanation for this correlation is the spatial heterogeneity of transmission within towns and cities. The latter, we have shown, cannot usefully be captured via the simple device of an exponent in the transmission term. More detailed explorations of disaggregated data and/or models with explicit spatial structure have the potential to shed light on this question. Finally, the strong evidence in favour of extra-demographic stochasticity raises the question of precisely why such stochasticity aids in the explanation of the data. To what extent does this finding indicate the presence of genuine environmental stochasticity? To what extent does it indicate model misspecification? To address these questions, again, analysis based on more detailed models is called for. In the case of measles, there are sufficient data to entertain models featuring spatial inhomogeneity (Grenfell *et al.* 1995; Xia *et al.* 2004; Bjørnstad & Grenfell 2008), age structure inhomogeneity (Schenzle 1984; Bolker & Grenfell 1996; Keeling & Grenfell 1997) or both (Bolker & Grenfell 1995). Although it has been demonstrated that such models can do a good job of accounting for gross features of the data, there has been less emphasis on requiring these models to account for all features of the data.

Progress on inference methodology for parameter estimation from measles time series has emphasized SEIR-type models for homogeneous populations (Ellner *et al.* 1998; Bjørnstad *et al.* 2002; Cauchemez & Ferguson 2008; Keeling & Ross 2008). The study of age structure and spatial effects for measles has placed less emphasis on inferring parameters from data (one exception is Xia *et al.* 2004). This may be partly because of the additional difficulties of inference for such systems and partly because models based on homogeneous mixing do an impressive job of describing key dynamic features (Earn *et al.* 2000; Grenfell *et al.* 2002). Regardless of one's view of the importance of paying further attention to population inhomogeneities, there is a natural methodological question: how applicable are the techniques presented here for larger, more complex models? There is a computational price to pay for the convenience of plug-and-play statistical methods. For the results in table 2, one application of the plug-and-play-iterated filtering inference procedure (based on 50 iterations, with a Monte Carlo size of 10^4^) implemented via the Pomp package in R (R Development Core Team 2006; King *et al.* 2009) took 5 h to run on a desktop machine. Computational effort scales roughly linearly with the number of parameters plus the number of state variables, so one can see that only a modest amount of additional structure could be included without hitting computational limitations. To increase computational efficiency, however, the iterated filtering algorithm can be implemented in a non-plug-and-play mode, in which the filter is either tailored to the particular model or takes advantage of available analytic properties of the transition densities. Such extensions, which would be required for much larger models, are a topic for future research. One can seek inspiration for future possibilities from numerical climate models, for which filtering operations (the computationally intensive step in the iterated filtering algorithm) have been carried out on systems with 5 × 10^6^ state variables (Anderson & Collins 2007). Filtering in such high-dimensional situations requires the development and evaluation of appropriate approximations for the system under investigation.

In this article, we have carried out the first scientific investigation based on a new framework for continuous-time, discrete-state population dynamics with both demographic and extra-demographic noise (probabilistic and statistical properties of this model class were investigated by Bretó *et al.* (2009). Extra-demographic stochasticity (interpreted as noise in the rates of a discrete population Markov population model) is equivalent to the possibility of multiple individuals moving simultaneously between compartments (Bretó *et al.* 2009), and, as such, may be due to social events that affect the behaviour of many individuals (e.g. sporting events) or events that change disease transmissibility, such as variations in temperature and humidity. Variability in the rates gives the model additional flexibility that can also describe model misspecification. Analogously, when carrying out linear regression, it is customary to fit a line to data while understanding that the variation of the data around the line corresponds to unknown and unmodelled deterministic effects as well as to random fluctuations. For linear regression, one typically treats both these sources of uncertainty equally, and certainly all the usual standard errors and test statistics do not discriminate between them. We maintain that the same approach can be applied to dynamic models; in other words, the distinction between model misspecification and process stochasticity should be noted, though it will not usually affect the subsequent analysis.

The term *extra-demographic stochasticity* encompasses all sources of variability beyond the intrinsic demographic stochasticity that would be present in a homogeneous population. There are many circumstances in which such variability can be expected to be important. In particular, the variability of rates in our new framework offers an approach to modelling superspreading events (Lloyd-Smith *et al.* 2005). These events occur when variability between individuals, environmental effects or an interaction between the two results in a highly skewed distribution for the number of secondary cases caused by an index case. Superspreading has been documented in measles, but is of greater dynamic importance in other diseases such as severe acute respiratory syndrome (Lloyd-Smith *et al.* 2005). Conventional population models amenable to non-plug-and-play statistical analyses have been unable to include such effects readily. This is, therefore, one more example in which the flexibility of plug-and-play methodology holds the potential to encourage the development of scientifically appropriate models.

## Appendix A. compartment models with stochastic rates

Compartment models provide a unifying framework to describe population dynamics (Jacquez 1996; Matis & Kiffe 2000). Here, we write down a general compartment model to obtain formal representations of the transition rates presented in §2. Specifically, we present a framework in which these rates can be interpreted as a system of ordinary differential equations, a discrete population stochastic process with demographic stochasticity or a discrete population stochastic process with both demographic and extra-demographic stochasticity.

A compartment model is a vector-valued process *X*(*t*) = (*X*_1_(*t*), … , *X*_*k*_(*t*)) denoting the (integer or real-valued) counts in each of *k* compartments labelled 1 to *k*. To keep track of individuals moving between compartments, we define flow processes *N*_*ij*_(*t*) such that *N*_*ij*_(*t*) – *N*_*ij*_(*s*) counts the number of transitions from compartment *i* to compartment *j* between times *s* and *t*. We introduce an extra compartment, labelled ∅, which provides a source and a sink to describe passages into and out of the system because of events such as birth, immigration, emigration and death. The basic characteristic of a compartment model is that *X*(*t*) can be written in terms of the flows *N*_*ij*_(*t*) from *i* to *j*, via a conservation of individuals' identity. Namely, for *i* ranging over 1, … ,*k* and *j* ranging over ∅, 1, … ,*k*, we write

A 1

Each *flow* *N*_*ij*_ is associated with a *rate* function . The corresponding per capita transition rate  is defined, for , by

A 2

There are many ways to develop concrete interpretations of such a compartment model (Bretó *et al.* 2009). For example, an ordinary differential equation interpretation is given by

for *i* ≠ *j* with *i* and *j* ranging over ∅, 1, … ,*k*. In this case, equation (A 1) becomes

A 3

This deterministic, continuous-population system is known as the deterministic skeleton (Cushing *et al.* 1998; Coulson *et al.* 2004). Continuous-population stochastic generalizations include stochastic differential equation models (Ionides *et al.* 2006; King *et al.* 2008) and ordinary differential equations with slowly varying rates (Swishchuk & Wu 2003).

A Markov chain interpretation can be specified by the infinitesimal transition probabilities (Brémaud 1999)

A 4

This Markov chain model includes only demographic stochasticity. Extra-demographic stochasticity is to be expected in data (Bjørnstad & Grenfell 2001); in particular, it arises because of variability in the rates of equation (A 4). We write *σ*_*ij*_(*t*, *X*(*t*)) for an intensity parameter corresponding to white noise in the rate ν_*ij*_(*t*, *X*(*t*)). Bretó *et al.* (2009) developed a general framework for adding white noise to the rates of Markov chain compartment models. The requirement that rates be non-negative rules out the use of Gaussian white noise; the simplest and most convenient parametric choice available is Gamma noise, which is employed in the Euler scheme of box 1. In box 1, *δ**Γ*_*ij*_ has dimension of time, so *δ**Γ*_*ij*_/*δ* is an approximation to multiplicative noise over a Euler time increment of duration *δ*. Box 1 reduces to a multinomial Euler scheme (Cai & Xu 2007) if all the noise parameters are set to zero.

For measles, a well-studied compartment model corresponds to *X*(*t*) = (*S*(*t*), *E*(*t*), *I*(*t*), *R*(*t*)). The state ∅ models both a source (corresponding primarily to births) and a sink (corresponding primarily to deaths); thus in §2 we write *N*_∅S_ = *N*_BS_, *N*_S∅_ = *N*_SD_, *N*_E∅_ = *N*_ED_, *N*_I∅_ = *N*_ID_ and *N*_R∅_ = *N*_RD_. The per capita recruitment rate *μ*_BS_(*t*) in §2 is given by

A 5

Here, *b*(*t*) is the birth rate, treated as known from annual birth data; *δ*(*t* − *t*_0_ mod 365) contributes a Dirac delta impulse to the recruitment rate when *t* falls on the same calendar day as *t*_0_, and we take *t*_0_ = 251 for the school admission day in England and Wales. The corresponding population recruitment rate is ν_*BS*_ = *μ*_*BS*_(*t*)*N*(*t*), where *N*(*t*) is the population size, treated as known via smooth interpolation of census data. The force of infection, *μ*_*SE*_(*t*), is given by equations (2.1) and (2.2). The calendar-day timings of the school holidays in equation (2.2) are as follows: Christmas, 356–365 and 0–6; Easter, 100–115; summer, 199–252; autumn half-term, 300–308.

Box 1. Euler scheme for a numerical solution of a Markov chain with stochastic rates. Here, (*α*, *β*) is the gamma distribution with shape parameter *α* and scale parameter is *β*, with corresponding mean *α**β* and variance *α**β*^2^. In the case *σ*_*ij*_ = 0, Gamma(*δ*/*σ*_*ij*_^2^, *σ*_*ij*_^2^) is defined to take the non-random value *δ*. Further details on the gamma, multinomial and Poisson distributions can be found in introductory texts on probability and statistics (Casella & Berger 1990). In steps 5 and 7, *R*_*i*_ counts the number of individuals who remain in compartment *i* during the current Euler increment.

- Set the initial value *X*(0) and time interval (0,*T*)
- Set time increment *δ*; define *t*_*n*_ = *n**δ* and *N* = *T*/*δ*.
- FOR *n* = 0 to *N* − 1
- Generate independent noise increments, *δ**Γ*_*ij*_ ∼ Gamma(*δ*/*σ*_*ij*_^2^,*σ*_*ij*_^2^).
- For *i* in 1, … ,*k*, generate process increments
  where *p*_*ij*_ = *p*_*ij*_({*μ*_*ij*_(*t*_*n*_, *X*(*t*_*n*_))},{*δ**Γ*_*ij*_}) is given by
  with *μ*_*ij*_ = *μ*_*ij*_(*t*, *x*), defined in equation (A 2).
- Generate process increments (*δ**N*_∅1_, … ,*δ**N*_∅*k*_) via
- Set *X*_*i*_(*t*_*n*+1_) = *R*_*i*_ + *σ*_*j*≠*i*_*δ**N*_*ji*_
- END FOR

To complete the model specification, we need to specify the measurement model. We write *Z*_*n*_ = *N*_IR_(*t*_*n*_)–*N*_IR_(*t*_*n*−1_) for the complete case count in the *n*th reporting interval, supposing that cases are quarantined once they are identified. The corresponding case report *y*_*n*_ is modelled via an overdispersed binomial distribution defined as a discretized normal random variable. Specifically, for *y* > 0, we take

A 6

where *Φ*(·; *μ*, *σ*^2^) is normal(*μ*,*σ*^2^) cumulative distribution function. For *y* = 0, we replace *y* − 0.5 by −∞ in equation (A 6). Here, *ρ* is the reporting rate and *ψ* the overdispersion parameter. Equation (A 6) represents a normal approximation to the binomial distribution with extra variability added. For *ψ*^2^≪1, which is the case for all communities of population greater than 10^5^ in the sample we investigated, the formulation in equation (A 6) approximates a binomial distribution for small *z* and a lognormal distribution for large *z*.

In the conventional SEIR Markov chain model given by equation (A 4), individuals move from *E* to *I* and then *I* to *R* at constant rates and so the latent and infectious periods are exponentially distributed. Gamma-distributed latent and infectious periods, which are believed to be more realistic, can be realized by dividing exposed and infectious classes into multiple stages (Anderson & Watson 1980; Lloyd 2001*a*,*b*). We implemented this and found a reduction in the estimates of *R*_0_ (as predicted by Wearing *et al.* 2005) but no substantial improvement in goodness of fit (figure S6, electronic supplementary material). In §2, we calculate *R*_0_ ignoring the negligible effects of mortality, taking *R*_0_ = *β̄*IP, where IP is the mean infectious period. Naïvely taking *IP* = *μ*_*IR*_^−1^ in box 1 produces a significant distortion when *μ*_*IR*_^−1^ is close to, or smaller than, the time discretization step *δ*. Throughout this work, we use *δ* = 1 day and we use the discrete-time formulas for IP and LP,

A 7
